# Deletion of ENTPD3 does not impair nucleotide hydrolysis in primary somatosensory neurons or spinal cord

**DOI:** 10.12688/f1000research.4563.2

**Published:** 2014-09-19

**Authors:** Eric McCoy, Sarah Street, Bonnie Taylor-Blake, Jason Yi, Martin Edwards, Mark Wightman, Mark Zylka

**Affiliations:** 1 Department of Cell Biology and Physiology, UNC Neuroscience Center, University of North Carolina, CB #7545, Chapel Hill, NC, 27599, USA; 2 Department of Chemistry, UNC Neuroscience Center, University of North Carolina, CB #3290, Chapel Hill, NC, 27599, USA

## Abstract

Ectonucleotidases are membrane-bound or secreted proteins that hydrolyze extracellular nucleotides.  Recently, we identified three ectonucleotidases that hydrolyze extracellular adenosine 5’-monophosphate (AMP) to adenosine in primary somatosensory neurons.  Currently, it is unclear which ectonucleotidases hydrolyze ATP and ADP in these neurons.  Ectonucleoside triphosphate diphosphohydrolases (ENTPDs) comprise a class of enzymes that dephosphorylate extracellular ATP and ADP.  Here, we found that ENTPD3 (also known as NTPDase3 or CD39L3) was located in nociceptive and non-nociceptive neurons of the dorsal root ganglion (DRG), in the dorsal horn of the spinal cord, and in free nerve endings in the skin.  To determine if ENTPD3 contributes directly to ATP and ADP hydrolysis in these tissues, we generated and characterized an *Entpd3* knockout mouse.  This mouse lacks ENTPD3 protein in all tissues examined, including the DRG, spinal cord, skin, and bladder.  However, DRG and spinal cord tissues from *Entpd3 ^-/-^* mice showed no reduction in histochemical staining when ATP, ADP, AMP, or UTP were used as substrates.  Additionally, using fast-scan cyclic voltammetry (FSCV), adenosine production was not impaired in the dorsal spinal cord of *Entpd3 ^-/-^* mice when the substrate ADP was applied.  Further, *Entpd3 ^-/- ^*mice did not differ in nociceptive behaviors when compared to wild-type mice, although *Entpd3 ^-/- ^*mice showed a modest reduction in β-alanine-mediated itch.  Taken together, our data indicate that deletion of *Entpd3* does not impair ATP or ADP hydrolysis in primary somatosensory neurons or in dorsal spinal cord.  Moreover, our data suggest there could be multiple ectonucleotidases that act redundantly to hydrolyze nucleotides in these regions of the nervous system.

## Introduction

Nucleotides like ATP are released from neurons and glia throughout the nervous system in response to physiological and pathological stimuli ( Arcuino *et al.*, 2002; Gourine *et al.*, 2010; Matsuka *et al.*, 2008; Nakamura & Strittmatter, 1996). Nucleotides signal through activation of the purinergic P2X and P2Y receptors and can excite or sensitize nociceptive neurons ( Burnstock, 2007; Dussor *et al.*, 2009; Sawynok, 2007; Tsuda *et al.*, 2005). The actions of extracellular ATP can be terminated by several membrane-bound and secreted ectonucleotidases that hydrolyze ATP into adenosine ( Sowa *et al.*, 2010b; Street *et al.*, 2013; Street *et al.*, 2011; Vongtau *et al.*, 2011; Zimmermann, 2006; Zylka *et al.*, 2008). Adenosine, in turn, can signal through the A _1_ adenosine receptor (A _1_R) to inhibit the activity of nociceptive neurons in the spinal cord ( Sawynok & Liu, 2003; Zylka, 2011).

**Figure 1.**
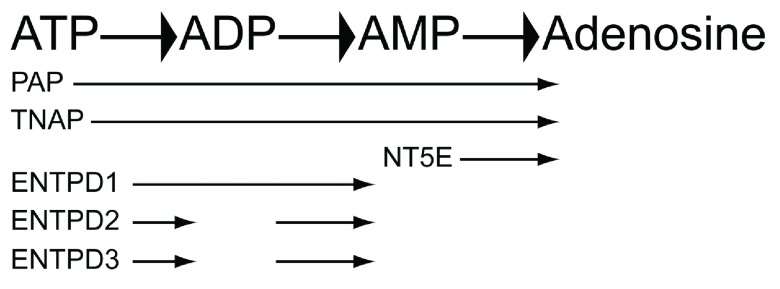
Ectonucleotidases, their substrates, and products. Several ectonucleotidases, depicted here, have been shown to hydrolyze adenosine-containing extracellular nucleotides such as ATP in a stepwise process into adenosine.

We previously identified and characterized the ectonucleotidases that hydrolyze AMP in nociceptive neurons ( Figure 1). These enzymes are Prostatic acid phosphatase (PAP; ( Street *et al.*, 2011; Zylka *et al.*, 2008)), Ecto-5’-nucleotidase (NT5E; ( Sowa *et al.*, 2010b; Street *et al.*, 2011)), and Tissue-nonspecific alkaline phosphatase (TNAP; ( Street *et al.*, 2013)). Pharmacological and knockout mouse model studies suggest that each of these enzymes contributes to the production of adenosine from AMP in the dorsal spinal cord, where nociceptive neurons synapse with spinal neurons ( Street *et al.*, 2013). Further, knockout mice lacking PAP, NT5E, or both PAP and NT5E showed enhanced nociceptive sensitization in models of chronic pain ( Sowa *et al.*, 2010b; Street *et al.*, 2011; Zylka *et al.*, 2008). While NT5E hydrolyzes AMP into adenosine (optimal activity at neutral pH), PAP (at neutral and acidic pHs) and TNAP (at basic pH) can also hydrolyze ATP, ADP, and AMP ( Ciancaglini *et al.*, 2010; Sowa *et al.*, 2009; Street *et al.*, 2013; Zimmermann, 2006). Others found that ectonucleoside triphosphate diphosphohydrolases (ENTPDs), an additional class of ectonucleotidases, might also be responsible for hydrolyzing ATP and ADP in primary somatosensory neurons ( Vongtau *et al.*, 2011).

In the ENTPD family, four (ENTPD1, -2, -3, and -8) are membrane-bound enzymes that hydrolyze extracellular ATP and ADP ( Robson *et al.*, 2006). ENTPD1, -2, and -3 are expressed throughout the central nervous system and display different preferences and kinetics for each nucleotide substrate ( Kukulski *et al.*, 2005; Langer *et al.*, 2007). The hydrolysis of ATP by ENTPD1 results in an increase in AMP levels, suggesting ENTPD1 rapidly hydrolyzes ATP and ADP substrates, whereas ENTPD2 preferentially dephosphorylates ATP, resulting in a buildup of extracellular ADP ( Figure 1). In contrast, ENTPD3 displays an intermediate activity between ENTPD1 and -2, showing rapid hydrolysis of ATP and transient increases in ADP before conversion into AMP ( Kukulski *et al.*, 2005). ENTPD1, -2, and -3 are expressed at similar levels in different cell types of the DRG and spinal cord ( Rozisky *et al.*, 2010; Vongtau *et al.*, 2011). Specifically, ENTPD1 is primarily expressed in blood vessels, ENTPD2 is primarily expressed in glial cells, including satellite cells and non-myelinating Schwann cells, and ENTPD3 is preferentially expressed in DRG neurons and their central and peripheral projections ( Braun *et al.*, 2004; Vongtau *et al.*, 2011). Further, ENTPD3 co-localizes with markers of nociceptive neurons, such as TRPV1, NT5E, and IB4-binding ( Vongtau *et al.*, 2011). These findings suggested that ENTPD3 might contribute to ATP and ADP hydrolysis in nociceptive neurons ( Vongtau *et al.*, 2011).

To study the contribution of ENTPD3 to ATP and ADP hydrolysis in nociceptive and non-nociceptive neurons in the DRG, we generated a knockout mouse that globally lacked ENTPD3 protein. As part of these studies, we performed immunohistochemical experiments to determine which subsets of DRG neurons expressed ENTPD3 and how loss of ENTPD3 altered nucleotide hydrolysis and nociceptive behaviors. Fast-scan cyclic voltammetry (FSCV) was used to examine adenosine generation in wild-type (WT) and *Entpd3* ^-/-^ mice. We found no significant differences between WT and *Entpd3 ^-/-^* mice in assays of ectonucleotidase function or in nociceptive behavioral assays, suggesting that additional enzymes are involved in the hydrolysis of ATP and ADP in nociceptive and non-nociceptive neurons.

## Methods

### Animal care and use

All vertebrate animals and procedures used in this study were approved by the Institutional Animal Care and Use Committee at the University of North Carolina at Chapel Hill. Mice were maintained on a 12 h:12 h light:dark cycle, were given food (Harlan 2920X) and water ad libitum, and were tested during the light phase. Mice were acclimated to the testing room, equipment and experimenter 1–3 days prior to testing.

### Molecular biology and knockout mouse generation

Recombineering was used to generate the *Entpd3* targeting arms from a 129S7/SvEv-derived bacterial artificial chromosome (BAC; bMQ-111o06; CHORI). The start codon, located in exon 2 ( Lavoie *et al.*, 2004), was replaced with an *AscI* site to facilitate cloning of *AscI*-LoxP-EGFPf-3xpA-LoxP-DTR-pA-Frt-PGK-NeoR-Frt- *AscI*. EGFPf=farnesylated enhanced GFP ( Zylka *et al.*, 2005), DTR=human diptheria toxin receptor ( Saito *et al.*, 2001). Use of this construct for axonal tracing and cell ablation of calcitonin gene-related peptide (CGRP)-expressing DRG neurons was previously described ( McCoy *et al.*, 2013; McCoy *et al.*, 2012). Correct targeting was confirmed in 5.2% of all embryonic stem cell clones by Southern blotting using flanking 5’ and 3’ probes and a NeoR internal probe. High percentage chimeras were crossed to C57BL/6 females to establish germline transmission and then crossed to PGK1-FLPo mice [B6(C3)-Tg(Pgk1-FLPo)10Sykr/J, Jackson Laboratory] to remove the Frt-flanked selection cassette (confirmed by PCR). Mice were backcrossed to C57BL/6 mice for eight generations to remove the PGK1-FLPo allele (confirmed by PCR) and establish the *Entpd3 ^-/-^* line. Note, the knocked-in GFP was undetectable in DRG and spinal cord neurons of the *Entpd3 ^-/-^* line.

### Immunoblotting

Male WT and *Entpd3 ^-/-^* (3 month-old; ~25 g; n=3 for each genotype) were decapitated, and the DRG and bladder tissue was collected and digested in modified RIPA buffer (50 mM HEPES pH 7.4, 150 mM NaCl, 1% Triton X-100, 1% SDS, 0.5% deoxycholate) supplemented with protease inhibitors (Roche Complete Mini). Protein samples from the cell lysate (1 mg/ml) were analyzed by SDS-PAGE with 4–20% gradient Tris-Glycine polyacrylamide gels (BioRad). The protein samples were then transferred to a polyvinylidene difluoride membrane and probed with a sheep anti-ENTPD3 polyclonal antibody overnight at 4°C (0.2 μg/ml; AF4464, R&D Systems), followed by a rabbit anti-sheep horseradish peroxidase-conjugated secondary antibody for 1 h at room temperature (0.16 μg/ml; #31480, Thermo Scientific).

### Tissue collection and preparation for histology

Hindpaw skin (glabrous and hairy), lumbar DRGs, and spinal cords were removed from male mice (n=3; ~10 weeks old) following decapitation, and immersion-fixed in cold 4% paraformaldehyde in 0.1 M phosphate buffer, pH 7.4, for 3 h, 4 h, and 8 h, respectively, and then cryoprotected in 30% sucrose in 0.1 M phosphate buffer at 4°C. DRGs were sectioned at 20 µm and collected on SuperFrost Plus slides; spinal cords and hindpaw skin were sectioned at 30 µm and 60 µm, respectively, and collected in PBS or a cryoprotectant solution containing PBS, ethylene glycol, and glycerol for long-term storage at -20°C.

### Histochemistry

Enzyme histochemistry was performed as described previously ( Zylka *et al.*, 2008) with a few modifications. Sections of DRG and spinal cord from 3 WT and 3 *Entpd3 ^-/-^* mice were incubated with a given concentration of a nucleotide (AMP, 6 mM for DRG, 3 mM for spinal cord; ADP, 1 mM for DRG and spinal cord; ATP, 0.2 mM for DRGs and spinal cord; UTP, 0.2 mM for spinal cord) in Trizma-maleate buffer containing 20 mM MgCl _2_, pH 7.0, and 2.4 mM lead nitrate for 3 h at room temperature. For some experiments, we included (in the rinse and substrate incubation steps) 10 mM levamisole to block alkaline phosphatase activity, 5 mM ouabain to block Na+/K+-ATPases, a combination of levamisole and ouabain, or 0.1–1.0 mM ARL67156 ( *N*-diethyl- d-β,γ-dibromomethylene ATP) to nonselectively block ENTPD enzymes. All reagents were purchased from Sigma.

### Immunohistochemistry

Tissue sections from 3 WT and 3 *Entpd3 ^-/-^* mice were stained immunohistochemically as previously reported ( Taylor-Blake & Zylka, 2010). Antibodies used were: polyclonal sheep anti-mouse ENTPD3/CD39L3 (skin, 1:75; DRG and spinal cord 1:400; AF4464, R&D Systems), monoclonal mouse anti-NeuN (1:250; MAB377, Millipore), polyclonal chicken anti-Prostatic acid phosphatase (1:4,000; PAP, Aves Labs), polyclonal rabbit anti-NF200 (1:800; N4142, Sigma), monoclonal mouse anti-NF200 (Clone RT97; MAB5262, Millipore), polyclonal rabbit anti-CGRP (1:150; BML-CA1134, Enzo Life Sciences), polyclonal sheep anti-CGRP (1:300; BML-CA1137, Enzo Life Sciences); polyclonal rabbit anti-PKCγ (1:800; sc-211, Santa Cruz), and polyclonal rat anti-PECAM1/CD31 (1:400; Clone MEC 13.3, 553370, BD Biosciences). IB4 conjugated with Alexa Fluor dyes and secondary antibodies conjugated with Alexa Fluor dyes were purchased from Invitrogen. DRAQ5 (Catalog # 4084) was purchased from Cell Signaling Technology. Stained sections were imaged on a Zeiss LSM 510 confocal microscope or a Zeiss LSM 710 confocal microscope.

### Fast-Scan Cyclic Voltammetry (FSCV)

Mouse sagittal spinal cord slices were prepared as described previously ( Street *et al.*, 2011). In brief, male mice aged 1–2 months old (~15 g; n=7 for each genotype) were anesthetized with urethane before decapitation, and the spinal cords were dissected and sectioned at 4°C in buffer that contained the following (in mM): 87 NaCl, 2.5 KCl, 1.25 NaH _2_PO _4_, 26 NaHCO _3_, 75 sucrose, 10 glucose, 1.5 ascorbic acid, 0.5 CaCl _2_, 7 MgCl _2_. The slices were then incubated for 45 minutes in artificial cerebrospinal fluid (ACSF), which contained the following (in mM): 125 NaCl, 2.5 KCl, 1.25 NaH _2_PO _4_, 26 NaHCO _3_, 25 glucose, 2.5 CaCl _2_, 1.5 MgCl _2_. All solutions were bubbled with 95%O _2_/5%CO _2_ for the duration of the dissection, incubation, and experiment steps.

FSCV monitoring of adenosine was performed as previously reported ( Street *et al.*, 2011), with the major difference being 100 µM ADP was used as the nucleotide substrate. Briefly, a disk-shaped carbon fiber microelectrode (Amoco) was inserted ( Cahill *et al.*, 1996), with the disk facing downwards, into the superficial dorsal horn. The potential of the microelectrode was scanned linearly at 400 V/s from -0.4 V to 1.5 V and back again once every 100 ms and was held at -0.4 V otherwise (all potentials versus Ag/AgCl). A micropipette inserted approximately 100 µm from the microelectrode was used to pressure-eject a bolus of 100 µM ADP using a Picospritzer ^®^ III (Parker Instrumentation, Pinebrook, NJ) (ejection parameters: 1 s, 20 PSI). The current was recorded for 5 ejections, 5 minutes apart, at the same location in each sample to obtain a mean response. The current was processed, as previously described ( Street *et al.*, 2011), using the background subtracted current at the voltammetric peak at ~1.0 V potential, which has been shown to be sensitive to adenosine and not to nucleotides, such as ATP, ADP, and AMP ( Swamy & Venton, 2007).

### Behavioral assays

For all behavioral assays, ~3 month-old male WT (n=10) and *Entpd3 ^-/-^* (n=10; all mice weighing ~26 g) mice were tested in each assay. Mice were acclimated to handling, testing rooms and facilities prior to testing, and the experimenter was blinded to the genotype of each animal. Heat sensitivity was measured by heating each hindpaw once per day using the Plantar Test apparatus (IITC) with a cut-off time of 20 s. For the tail immersion assay, each mouse was gently restrained in a towel, and the distal one-third of the tail was immersed into a water bath heated to 46.5°C or 49°C or into 75% ethanol cooled to -10°C ( Wang *et al.*, 1995). The latency to flick or withdraw the tail was measured once per mouse. The cut-off was set at 40 s, 30 s, and 60 s, respectively. For the hot plate test, the latency to jump, shake, or lick a hindpaw was measured within a 30 s cut-off time. To determine mechanical sensitivity, we used an electronic von Frey apparatus (IITC) with semi-flexible tips. Two measurements from each hindpaw were taken and averaged to determine the paw withdrawal threshold in grams. The tail clip assay (noxious mechanical) and cotton swab assay (innocuous mechanical) were performed as described ( Garrison *et al.*, 2012; Lariviere *et al.*, 2002). For the acetone test ( Bautista *et al.*, 2007), each mouse was placed into a Plexiglas chamber with a wire mesh floor, 50 μL of acetone was placed onto the left hindpaw, and the time spent licking was measured for 1 minute. The cold plantar assay was performed with mice resting on the glass surface of the Plantar Test apparatus (IITC) ( Brenner *et al.*, 2012). For the two-temperature discrimination assay, each mouse was placed into a Plexiglas chamber covering two metal surfaces that could be set at different temperatures ( Bautista *et al.*, 2007; Dhaka *et al.*, 2007). The amount of time mice spent on each side over a 10 minute period was recorded. Hot and cold sensitivity was assessed on a metal plate heated/cooled to a range of temperatures (5–55°C), with a cut-off time of 30 s, as described ( Gentry *et al.*, 2010). For measuring itch responses, histamine (10 μg/μL), chloroquine (CQ; 4 μg/μL) or β-alanine (20 μg/μL) dissolved in 0.9% saline was injected subcutaneously into the nape of the neck (50 μL injection volume). The number of scratching bouts was measured for 30 minutes in 5 minute blocks. One bout consisted of a set of scratches at the injection site until the hindpaw was either licked or placed onto the floor. For the water repulsion assay ( Westerberg *et al.*, 2004), the mouse was immersed in a 37°C water bath for 2 min. The mouse was removed from the water and placed onto a paper towel for 5 s, then weight and rectal temperature (deep body temperature, Tb, measured using a digital thermometer, Acorn Temp TC Thermocouple) were measured every 5 min for 60 min. The Complete Freund’s adjuvant (CFA) model of inflammatory pain and the lysophosphatidic acid (LPA) model of neuropathic pain were performed as described ( Sowa *et al.*, 2010a; Zylka *et al.*, 2008). Twenty microliters of CFA was injected into the left hindpaw centrally beneath the glabrous skin, and 5 nmol of LPA was administered intrathecally.

## Data analysis

Data analysis was performed in Excel (version 2010) using t-tests for all behavioral studies and cell counts with all graphs created in GraphPad Prism. The FSCV data were analyzed using the analysis portion of the freely available software HDCV (Version 4). The software is available for download from: http://www.chem.unc.edu/facilities/index.html?display=electronics&content=software. Average peak currents from the FSCV data were compared using paired t-test. Significance was determined as p ≤ 0.05.

## Results and discussion

Influence of ENTPD3 deletion on nucleotide hydrolysis in mouse primary somatosensory neurons and spinal cord: data Please see the ‘Legends’ file in the zip file for descriptions of each data set.

### ENTPD3 colocalizes with nociceptive and non-nociceptive neuronal markers in DRG

**Figure 2.**
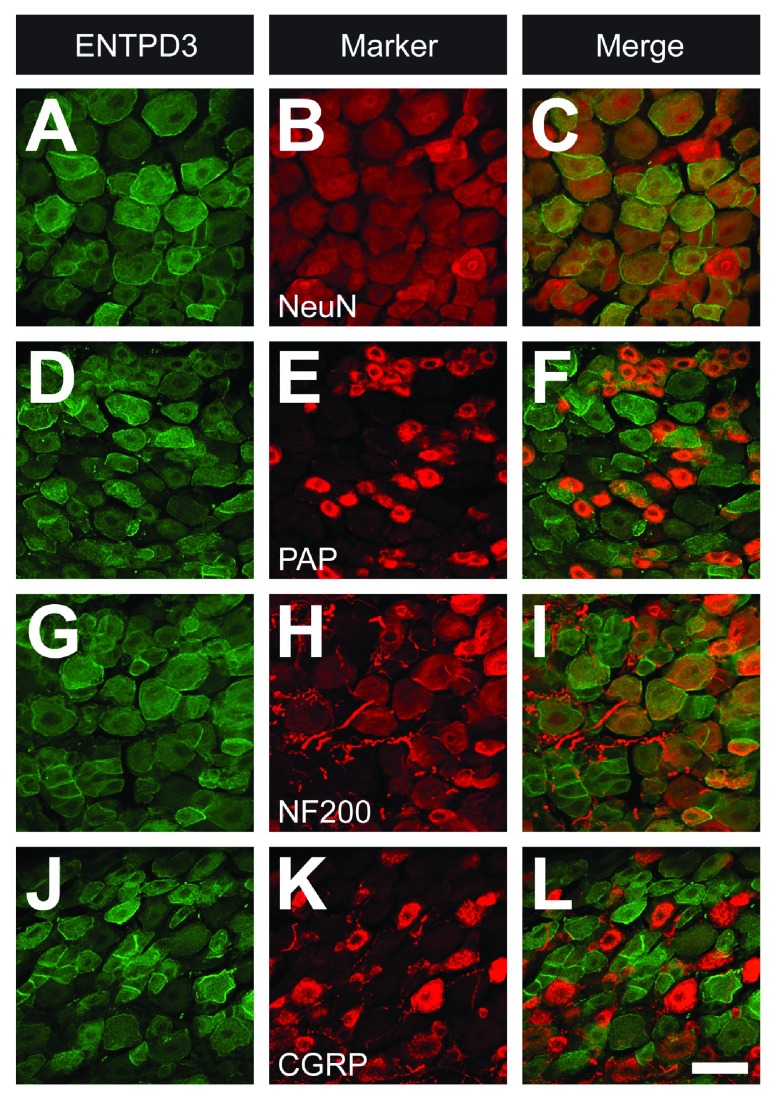
ENTPD3 is broadly expressed in DRG neurons. Mouse DRG neurons were immunostained for ENTPD3 ( **A**, **D**, **G**, **J**) and selected markers ( **B**, **E**, **H**, **K**). ( **C**, **F**, **I**, **L**) Merged images. Images were acquired by confocal microscopy. Scale bar: (in **L**) **A**– **L** = 50 μM.

ENTPD3 is expressed throughout the nervous system, including nociceptive neurons ( Belcher *et al.*, 2006; Langer *et al.*, 2007; Vongtau *et al.*, 2011). To determine which subsets of lumbar DRG neurons expressed ENTPD3, we immunostained for ENTPD3 and markers of nociceptive and non-nociceptive neurons. As previously reported ( Vongtau *et al.*, 2011), most DRG neurons, including small-, medium-, and large-diameter neurons, showed some level of staining for ENTPD3 ( Figure 2). For colocalization studies, we assessed only those neurons that were stained moderately to strongly for ENTPD3. All cells that expressed ENTPD3 also expressed NeuN, recapitulating previous results showing that ENTPD3 was primarily associated with neuronal cell types ( Figure 2A–C, Table 1) ( Belcher *et al.*, 2006; Langer *et al.*, 2007; Vongtau *et al.*, 2011). Conversely, 56.8% of all DRG neurons (identified by NeuN expression) labeled for ENTPD3 ( Figure 2A–C, Table 1). PAP, a marker of nonpeptidergic and some peptidergic nociceptive neurons, was extensively colocalized with ENTPD3—the majority (72.7%) of DRG neurons expressing PAP also expressed ENTPD3, while almost half (43.5%) of all ENTPD3 ^+^ neurons expressed PAP ( Figure 2D–F, Table 1). These results were similar to those found by Vongtau and co-workers, who reported that 97% of IB4-binding nonpeptidergic DRG neurons expressed ENTPD3 ( Vongtau *et al.*, 2011). NF200, a marker for large-diameter, non-nociceptive neurons and smaller, thinly myelinated (Aδ) nociceptive neurons, colocalized with ENTPD3 ( Figure 2G–I, Table 1), suggesting that ENTPD3 was expressed by some non-nociceptive neurons. Finally, an antibody to CGRP was used to identify peptidergic neurons ( Figure 2J–L). Of CGRP-expressing neurons, 48.7% were also positive for ENTPD3 ( Table 1). Thus, our results indicate that ENTPD3 is expressed in nociceptive and non-nociceptive neurons of the DRG.

**Table 1.** Analysis of co-expression of ENTPD3 and markers in WT DRG.

	Percentage of ENTPD3 ^+^ neurons expressing indicated marker	Percentage of marker ^+^ neurons expressing ENTPD3
**NeuN**	**100%** *(3,706 ENTPD3 ^+^ neurons evaluated)*	**56.8 ± 2.4** *(6,706 NeuN ^+^ neurons evaluated)*
**PAP**	**43.5 ± 2.1** *(3,860 ENTPD3 ^+^ neurons evaluated)*	**72.7 ± 1.4** *(2,107 PAP ^+^ neurons evaluated)*
**NF200**	**43.3 ± 2.3** *(3,706 ENTPD3 ^+^ neurons evaluated)*	**60.9 ± 2.3** *(2,589 NF200 ^+^ neurons evaluated)*
**CGRP**	**19.9 ± 0.9** *(3,860 ENTPD3 ^+^ neurons evaluated)*	**48.7 ± 3.2** *(1,657 CGRP ^+^ neurons evaluated)*

n=3 animals per genotype; 5 sections per animal. Values represent pooled data from each genotype.

### ENTPD3 expression in spinal dorsal horn

**Figure 3.**
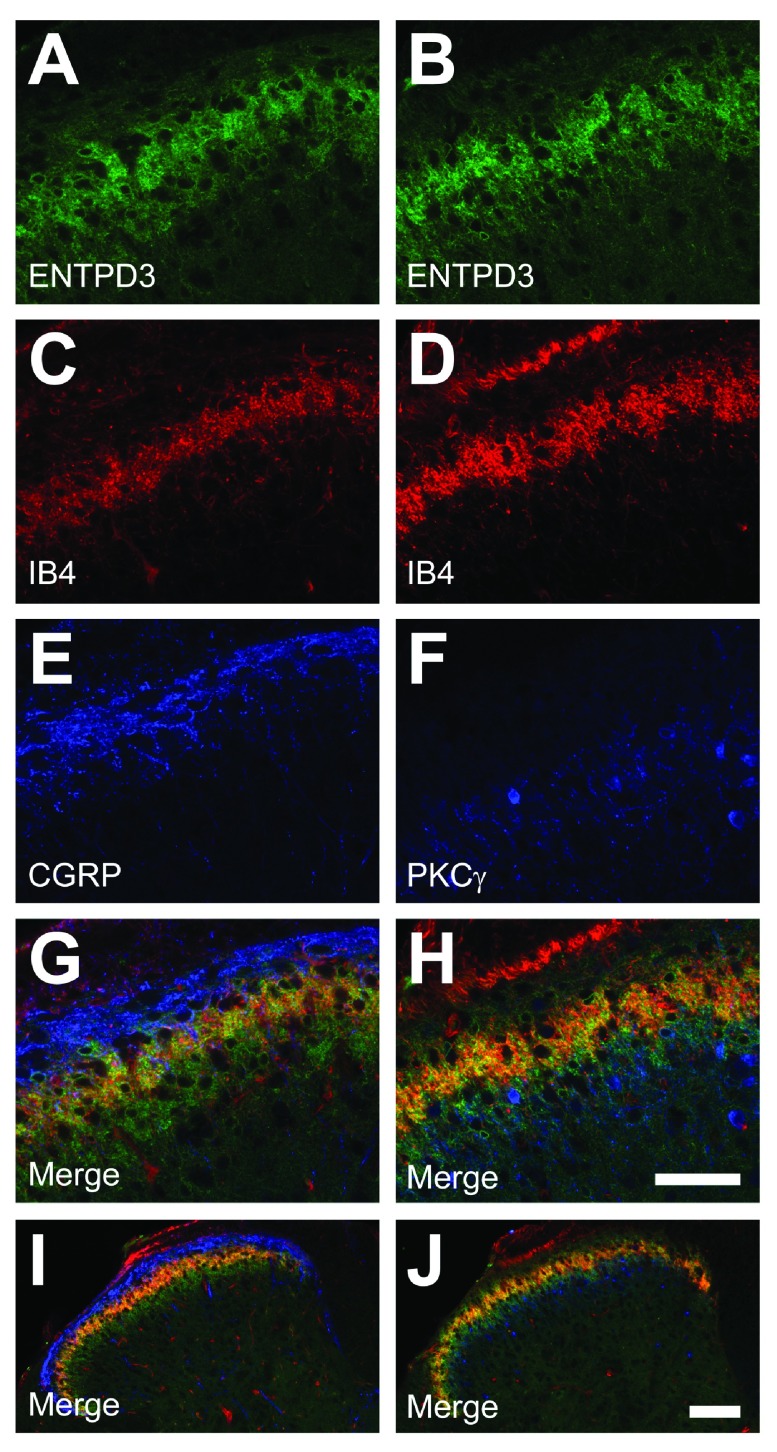
ENTPD3 is colocalized with peptidergic and nonpeptidergic markers in the dorsal horn of the spinal cord. Mouse lumbar spinal cord sections were immunostained for ENTPD3 ( **A**– **B**), and for the indicated markers ( **C**– **F**). ( **G**– **J**) Merged images at high ( **G**– **H**) and low ( **I**– **J**) magnification. Scale bar in **H** ( **A**– **H**) = 50 μM. Scale bar in **J** ( **I**– **J**) = 100 μM.

We also immunostained lumbar spinal cord sections to ascertain where ENTPD3 was located in the dorsal horn, the spinal region where axons of nociceptive and non-nociceptive sensory neurons terminate. ENTPD3 ^+^ nerve terminals were located primarily in lamina II, where IB4 terminals are located ( Figure 3A–D,I), consistent with a previous report ( Vongtau *et al.*, 2011). ENTPD3 ^+^ terminals also extended dorsally into lamina I, an area occupied by CGRP ^+^ terminals ( Figure 3E,G,I) and ventrally into lamina III, an area with Protein Kinase Cγ (PKCγ)-expressing spinal neurons ( Figure 3F,H,J). We also observed small ENTPD3 ^+^ spinal neurons in laminae I, II, and III ( Figure 3A,B,G,H) as was reported by Vongtau and co-workers ( Vongtau *et al.*, 2011). This localization pattern in spinal laminae and spinal neurons suggests that ENTPD3 might hydrolyze extracellular nucleotides in spinal pathways devoted to nociception and somatosensation.

### Generation and characterization of an *Entpd3 ^-/-^* mouse

**Figure 4.**
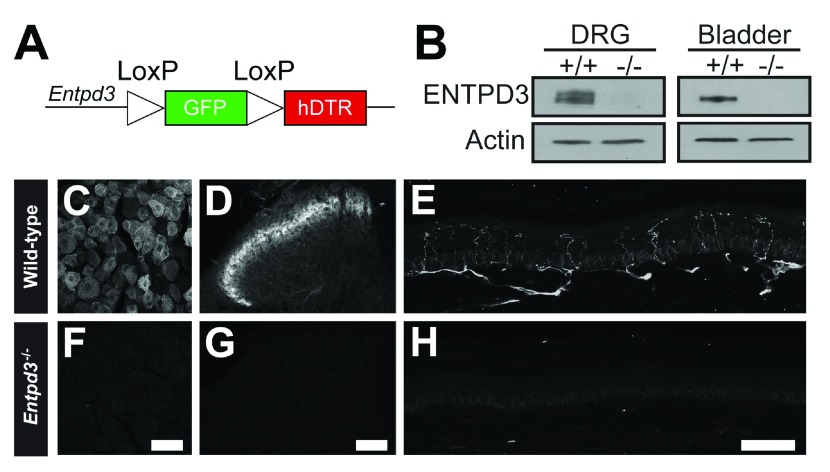
ENTPD3 protein is eliminated in *Entpd3 ^-/-^* mice. ( **A**) Cartoon depiction of the farnesylated GFP-DTR construct that was knocked-in to the start codon of *Entpd3*. ( **B**) Immunoblot showing loss of ENTPD3 protein in DRG and bladder collected from adult mice. ( **C**– **H**) Loss of ENTPD3 in DRG ( **F**), spinal cord ( **G**), and glabrous skin ( **H**) in *Entpd3 ^-/-^* mouse tissue compared to wild-type tissues ( **C**, **D**, and **E**, respectively). Scale bars: **D** = 50 μM; **F** = 100 μM; **H** = 50 μM.

To assess the extent to which ENTPD3 was necessary for extracellular nucleotide hydrolysis, we disrupted the *Entpd3* gene by knocking a LoxP-flanked GFP construct into the start codon of ENTPD3 ( Figure 4A). Expression of GFP was not detectable in DRG or spinal cord even when amplified with antibodies against GFP (image not shown). We were thus unable to use GFP to mark cells that expressed *Entpd3*. Using immunoblotting, we detected ENTPD3 protein in DRG and bladder (tissues known to express high levels of ENTPD3 ( Vongtau *et al.*, 2011; Yu *et al.*, 2011)) from WT mice, but no ENTPD3 protein was detectable in tissues from *Entpd3 ^-/-^* mice ( Figure 4B). These results confirmed that ENTPD3 protein was eliminated in our knockout line and that the antibody we used was specific for ENTPD3. We also immunohistochemically stained DRG, spinal cord, and hindpaw skin of WT and *Entpd3 ^-/-^* mice. We found that lumbar DRG sections from WT mice showed neuronal staining characteristic of ENTPD3, whereas sections from *Entpd3 ^-/-^* mice showed no staining ( Figure 4C,F). Similarly, sections of lumbar spinal cord and hindpaw skin from *Entpd3 ^-/-^* mice showed none of the ENTPD3 ^+^ neural profiles observed in WT spinal cord and hindpaw skin ( Figure 4D–E,G–H). Mice lacking ENTPD3 produced normal-sized litters (5–9 pups/litter) and had normal weights relative to WT mice (at 3 months ~26 g WT; ~27 g *Entpd3 ^-/-^*).

**Figure 5.**
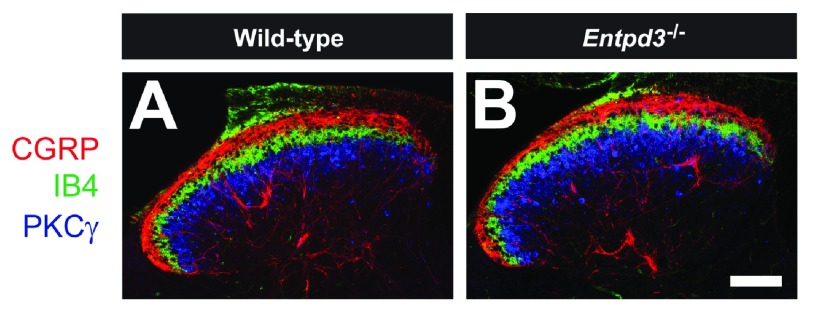
Superficial dorsal horn organization in *Entpd3 ^-/-^* mice is normal. Sections of lumbar spinal cord from WT ( **A**) and *Entpd3* ^-/-^ ( **B**) mice were stained with antibodies to distinguish laminar organization of the superficial dorsal horn. Scale bar in **B** = 100 μM.

Next, we used immunohistochemistry to determine if primary somatosensory neurons or axon terminals were affected by deletion of *Entpd3*. In DRG, the number of neurons that expressed nociceptive and non-nociceptive markers was not changed with the exception of a small, but statistically significant decrease in the number of neurons expressing NT5E ( Table 2). In WT mice, 35% of DRG neurons expressed NT5E, but in *Entpd3* ^-/-^ animals this percentage was reduced to 30.5% ( Table 2). We also used immunohistochemistry to assess whether the spinal dorsal horn of *Entpd3 ^-/-^* mice exhibited altered organization in comparison with that of WT animals. The laminar organization in the dorsal spinal cord of *Entpd3 ^-/-^* mice, as revealed by staining for CGRP and PKCγ and binding of IB4, was indistinguishable from that of WT mice ( Figure 5), suggesting that there was no alteration in the organization of primary afferents or spinal neurons in the dorsal horns of mice that lack ENTPD3.

**Table 2.** Marker analysis in WT and *Entpd3 ^-/-^* DRG sections.

	WT	*Entpd3 ^-/-^*	p value
**PAP**	40.6% ± 2.0 (6,900 neurons evaluated)	42.0% ± 1.8 (6,655 neurons evaluated)	0.61
**NF200**	39.8% ± 1.7 (6,706 neurons evaluated)	37.1% ± 2.2 (7,574 neurons evaluated)	0.33
**NT5E**	35.2% ± 1.2 (7,680 neurons evaluated)	30.5% ± 1.1 (8,520 neurons evaluated)	0.0054
**IB4**	32.0% ± 0.9 (6,828 neurons evaluated)	33.0% ± 1.3 (7,008 neurons evaluated)	0.55
**CGRP**	30.9% ± 1.3 (6,828 neurons evaluated)	27.6% ± 1.2 (7,008 neurons evaluated)	0.079

n=3 animals per genotype; 6 sections evaluated per animal. Values represent pooled data from each genotype.

**Figure 6.**
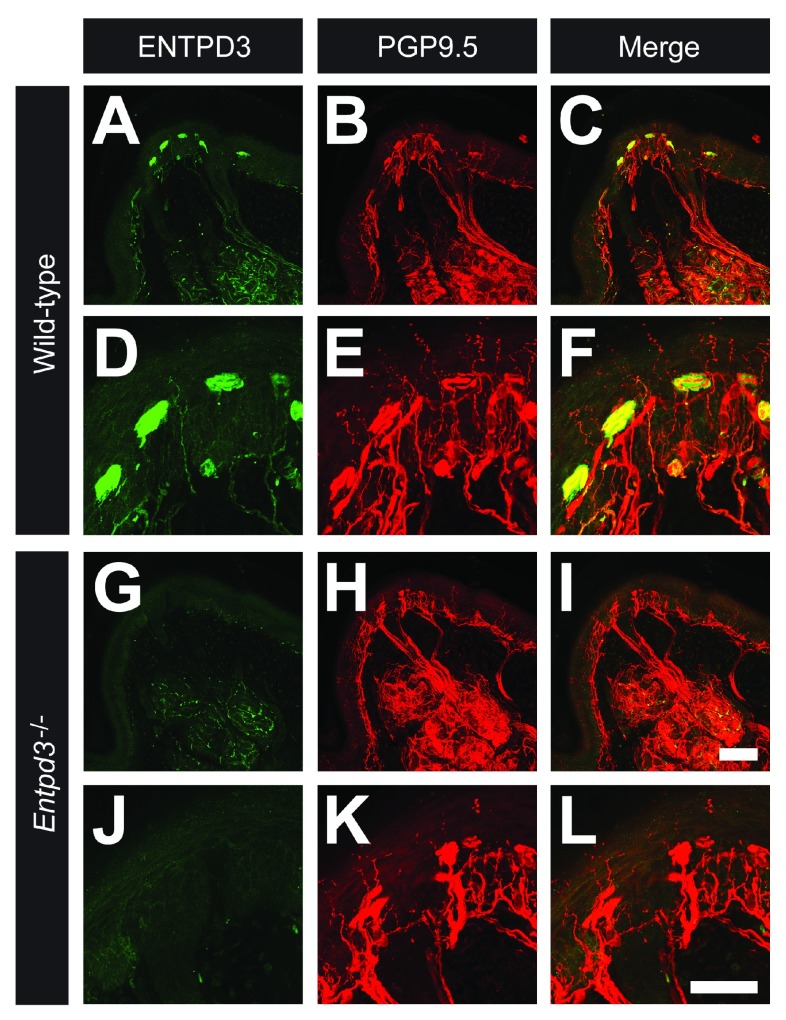
Deletion of *Entpd3* does not affect the density of nerve fiber staining in skin. Sections of glabrous skin from the volar pads in WT ( **A**– **F**) and *Entpd3 ^-/-^* ( **G**– **L**) mice were stained with antibodies against ENTPD3 ( **A**, **D**, **G**, **J**) and PGP9.5 ( **B**, **E**, **H**, **K**). **D**– **F** and **J**– **L** are high magnification insets of **A**– **C** and **G**– **H** and show Meissner corpuscles. Scale bar in **I** ( **A**– **C** and **G**– **I**) = 100 μM. Scale bar in **L** ( **D**– **F** and **J**– **L**) = 50 μM.

Finally, to determine if cutaneous innervation was altered in *Entpd3 ^-/-^* mice, we co-stained sections of glabrous and hairy skin of WT and *Entpd3 ^-/-^* mice with antibodies to ENTPD3 and PGP9.5, a pan-neuronal marker ( Figure 6). ENTPD3 marked most PGP9.5 ^+^ epidermal free nerve endings in hairy and glabrous skin as well as Meissner corpuscles and Merkel cells in volar pads ( Figure 6A–F). These findings were similar to the previously reported staining pattern of ENTPD3 in skin sections ( Vongtau *et al.*, 2011). Sections of skin from *Entpd3 ^-/-^* mice lacked all ENTPD3 staining. Expression of PGP9.5 was retained, revealing no differences in the density or structure of free nerve endings, Meissner corpuscles, and Merkel cells in *Entpd3 ^-/-^* mice compared to those observed in skin from WT mice ( Figure 6G–L). Thus, cutaneous innervation was not altered by the loss of ENTPD3. Further, nerve fibers co-expressing ENTPD3 and PGP9.5 were found on blood vessels in the dermis and deep dermis of the hindpaw (image not shown). There was no difference in the density of innervation of blood vessels (as revealed by PGP9.5 immunostaining) between WT and *Entpd3 ^-/-^* mice (image not shown). Taken together, these results suggest that, with the exception of a small decrease in NT5E staining in DRG neurons, deletion of *Entpd3* did not affect afferents in the skin, DRG neurons, or primary somatosensory afferents in the dorsal spinal cord.

### *Entpd3 ^-/-^* mice do not exhibit deficits in nucleotide hydrolysis or adenosine generation

**Figure 7.**
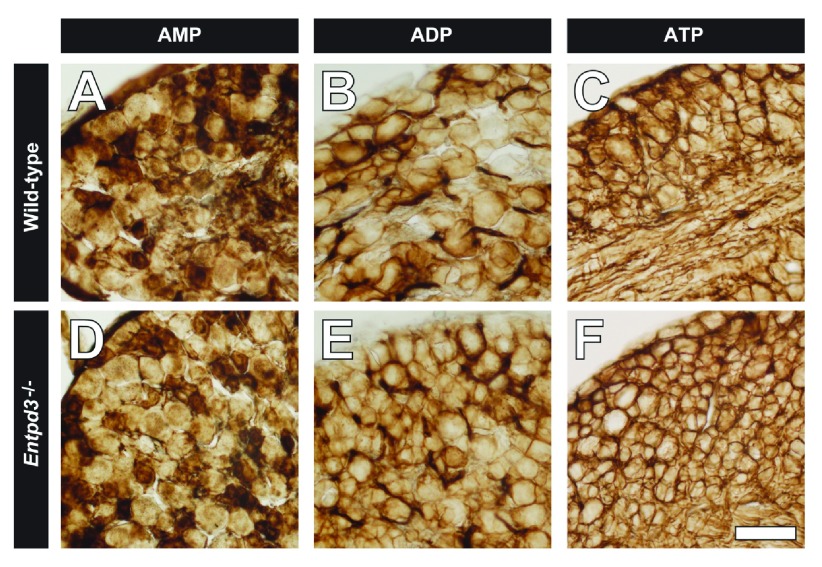
Nucleotide hydrolysis, assessed via enzyme histochemistry, is not reduced in DRG neurons from *Entpd3 ^-/-^* mice. DRG sections from WT ( **A**– **C**) and *Entpd3 ^-/-^* ( **D**– **F**) mice were stained using AMP ( **A**, **D**), ADP ( **B**, **E**), and ATP ( **C**, **F**) enzyme histochemistry at pH 7.0 in the presence of 20 mM MgCl _2_. Concentration of nucleotides used for histochemistry were as follows: AMP, 6 mM; ADP, 1 mM; ATP, 0.1 mM; UTP, 0.1 mM. Scale bar in **F** = 50 μM.

We previously reported that AMP hydrolysis in the DRG and dorsal spinal cord was redundantly carried out by three ectonucleotidases, PAP, NT5E, and TNAP ( Street *et al.*, 2013). However, the enzymes that contribute to ATP and ADP hydrolysis in DRG and spinal cord have not yet been fully characterized. To determine if ENTPD3 contributed to nucleotide hydrolysis in DRG, we performed histochemistry at a neutral pH (7.0) on DRG sections from WT and *Entpd3 ^-/-^* mice using the indicated nucleotides ( Figure 7). AMP histochemical staining was found in cell bodies of small- and medium-diameter neurons ( Figure 7A,D; where PAP and NT5E are located); ADP histochemical staining was strongest in blood vessels (where ENTPD1 is located) and on the membrane of most neurons ( Figure 7B,E); and ATP histochemical staining was present on blood vessels and the cell membrane of most neurons ( Figure 7C,F). These staining patterns matched what was previously seen in DRG sections from WT mice ( Sowa *et al.*, 2010b; Street *et al.*, 2011; Vongtau *et al.*, 2011; Zylka *et al.*, 2008).

When comparing staining between WT and *Entpd3 ^-/-^* DRGs, we saw no difference in AMP histochemical staining ( Figure 7A,D), consistent with the fact that AMP is not a substrate for ENTPD3 ( Ciancaglini *et al.*, 2010). Surprisingly however, there were also no differences in histochemical staining between WT and *Entpd3 ^-/-^* DRGs when ADP or ATP was used as substrates ( Figure 7B–C,E–F). These data suggest either that ENTPD3 does not hydrolyze these nucleotides in DRG or that other ADP- and ATP-hydrolyzing ectonucleotidases are present and function redundantly with ENTPD3. To determine if ENTPD3 hydrolyzed ADP and ATP redundantly with alkaline phosphatases at pH 7.0, we inhibited alkaline phosphatase activity in histochemical experiments with levamisole (10 mM). However, we observed no difference in staining between WT and *Entpd3 ^-/-^* DRGs in the presence of levamisole (image not shown). These data suggest DRG neurons contain additional ectonucleotidases besides TNAP and ENTPD3 that hydrolyze ATP and ADP at neutral pH.

**Figure 8.**
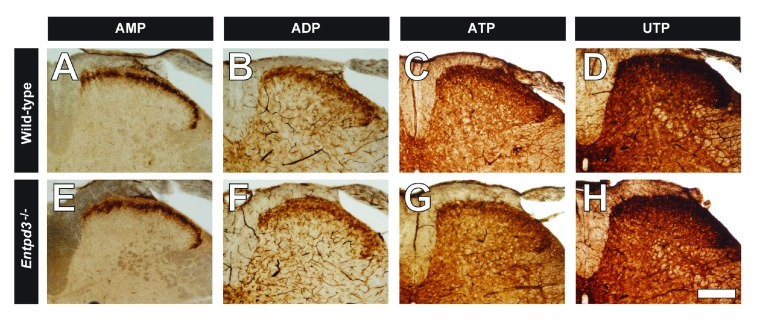
Nucleotide hydrolysis is not reduced in spinal cord sections from *Entpd3 ^-/-^* mice. Lumbar spinal cord sections from WT ( **A**– **D**) and *Entpd3 ^-/-^* ( **E**– **H**) mice were stained using AMP ( **A**, **E**), ADP ( **B**, **F**), ATP ( **C**, **G**), and UTP ( **D**, **H**) enzyme histochemistry at pH 7.0 in the presence of 20 mM MgCl _2_. Nucleotide concentrations were as follows: AMP, 3 mM; ADP, 1 mM; ATP, 0.2 mM; UTP, 0.2 mM. Scale bar in H = 200 μM.

**Figure 9.**
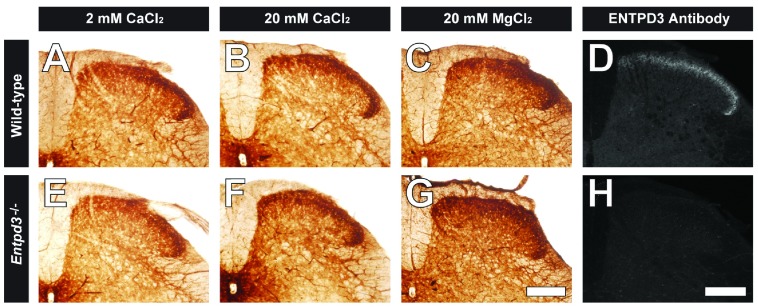
UTP hydrolysis in spinal cord sections from WT and *Entpd3 ^-/-^* mice is unaffected by substitution of divalent cations in histochemistry procedure. Lumbar spinal cord sections from WT ( **A**– **C**) and *Entpd3 ^-/-^* ( **E**– **G**) mice were examined for UTP hydrolysis at pH 7.0 in the presence of either 2 mM CaCl _2_ ( **A**, **E**), 20 mM CaCl _2_ ( **B**, **F**), or 20 mM MgCl _2_ ( **C**, **G**). UTP concentration for **A**– **B** and **E**– **F** is 0.2 mM and 0.1 mM for **C** and **G**. Representative images of ENTPD3 antibody staining in lumbar spinal cords from the same WT and *Entpd3 ^-/-^* mice are shown in **D** and **H**. Scalebar in **G**, **H** = 200 μM.

We also found that enzyme histochemical staining was equivalent in the superficial dorsal spinal cord of WT and *Entpd3 ^-/-^* mice when the indicated nucleotides were used as substrates ( Figure 8). Many ectonucleotidases, including ENTPD3 ( Lavoie *et al.*, 2004), are slightly more active in biochemical assays with calcium as the divalent cation. However, we detected no difference in UTP histochemical activity in spinal cord between WT and *Entpd3 ^-/-^* mice when 2 mM or 20 mM CaCl _2_ was substituted for 20 mM MgCl _2_ ( Figure 9; with deletion of ENTPD3 confirmed in these sections using immunostaining, Figure 9H). Thus Mg ^2+^ and Ca ^2+^ appear to be interchangeable in this histochemical assay.

To determine if other enzymes contributed to histochemical staining in the dorsal spinal cord when ATP and UTP (0.2 mM) were used as substrates, we used levamisole to block activity of alkaline phosphatases (10 mM), ouabain to block activity of Na ^+^/K ^+^-ATPase (5 mM), and ARL67156 (0.1 and 1 mM), an inhibitor of ENTPD1 and ENTPD3 ( Levesque *et al.*, 2007). The addition of these inhibitors did not result in any change in the staining intensity or pattern in the superficial dorsal horn of WT mice relative to *Entpd3 ^-/-^* mice, but adding ARL67156 caused a near-complete loss of histochemical staining in microglia in the spinal gray in both genotypes, presumably because of blockade of ENTPD1 activity ( Braun *et al.*, 2000) (image not shown). Vongtau *et al.* also tested various inhibitors (ouabain, levamisole, and sodium azide) to block Na ^+^/K ^+^-ATPase, alkaline phosphatase, and ENTPD1 activity, respectively ( Vongtau *et al.*, 2011). They found that none of these inhibitors affected ATP or UTP hydrolysis in the spinal cord and concluded that ENTPD3 might be responsible for the remaining staining. Our study demonstrates that the level of nucleotide histochemical staining was the same in the *Entpd3 ^-/-^* mice in the presence of ouabain and levamisole plus an ENTPD1/3 inhibitor (ARL67156), suggesting that one or more enzymes other than ENTPD3 are present that hydrolyze nucleotides in the spinal cord.

**Figure 10.**
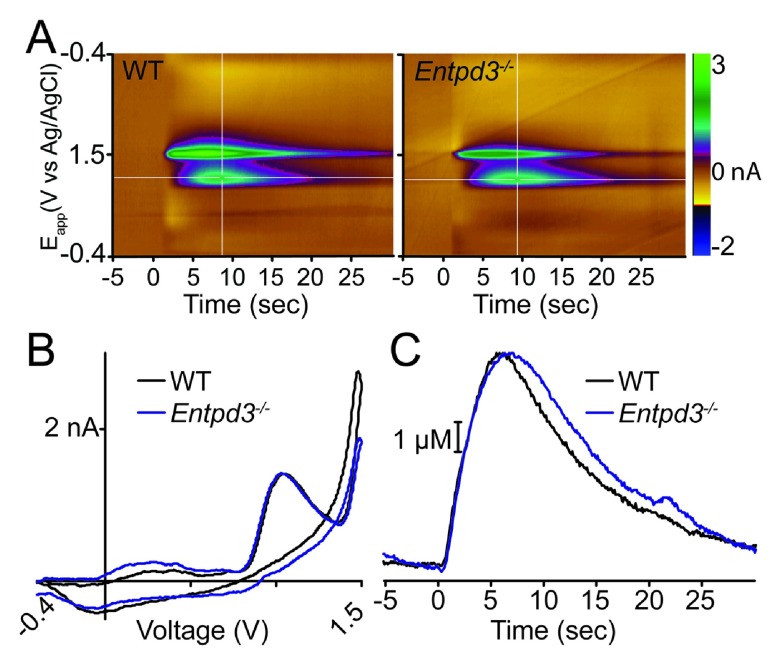
Hydrolysis of ADP to adenosine is not reduced in *Entpd3 ^-/-^* mice. FSCV was used to measure adenosine production in response to a bolus of ADP. ( **A**) Representative FSCV color plots: 100 μM of ADP was pressure ejected for 1 s onto lamina II of ( **A**) WT or *Entpd3 ^-/-^* mice. ( **B**) Cyclic voltammogram of one voltage trace from -0.4 V to 1.5 V and back to -0.4 V confirms the production of adenosine in WT (black trace) and *Entpd3 ^-/-^* animals (blue trace) (shown as an increase in current at 1.0 and 1.5 V). ( **C**) Adenosine concentration calculated from 1.0 V current (white horizontal lines in **A**) in WT (black trace) and *Entpd3 ^-/-^* (blue trace) animals. There was no statistically significant difference between slices from WT and *Entpd3 ^-/-^* mice (n=5 slices for each condition; paired t-test).

Enzyme histochemistry detects phosphate that is produced following nucleotide hydrolysis. As an alternative, we used FSCV to quantify adenosine production upon nucleotide hydrolysis in spinal cord slices of WT and *Entpd3 ^-/-^* mice. As previously reported, FSCV can be used to detect adenosine based on characteristic oxidation voltages at 1.0 and 1.5 V ( Swamy & Venton, 2007). We applied 100 mM ADP to lamina II and then measured adenosine production at the tip of a carbon-fiber microelectrode ( Street *et al.*, 2011). Application of ADP led to the generation of adenosine in WT and *Entpd3 ^-/-^* mice, detected as an increase in measured current at oxidation voltages of 1.0 and 1.5 V ( Figure 10A–B). Currents at 1.0 V were then converted to adenosine concentration. We then compared the peak adenosine concentration in WT and *Entpd3 ^-/-^* mice (n=5 slices/genotype) to determine if mice lacking ENTPD3 had any deficit in the production of adenosine ( Figure 10C). We saw no significant differences in adenosine generation from ADP between spinal cord slices of WT and *Entpd3 ^-/-^* mice.

Note that FSCV cannot resolve neuronal ENTPD3 activity in the dorsal horn from spinal microglial ENTPD1 activity, so the adenosine detected by FSCV after applying ADP could originate from microglial ENTPD1 or other ectonucleotidases in the tissue. For example, this adenosine could originate from PAP and/or TNAP, as these enzymes are located in the same region and can also hydrolyze ADP to adenosine ( Figure 1).

These FSCV results, when combined with enzyme histochemistry results, suggest that there are multiple ectonucleotidases that function redundantly to dephosphorylate ATP and ADP in DRG and superficial dorsal horn. Determining the molecular identities of these enzymes will require future studies with additional ectonucleotidase knockout mice and pharmacological inhibitors. Intriguingly, a redundant group of enzymes mediates AMP hydrolysis in the spinal cord, as PAP, NT5E, and TNAP must all be inhibited to completely block the generation of adenosine from AMP ( Street *et al.*, 2013). Likewise, TNAP can fully compensate for the loss of NT5E and generate adenosine from nucleotides in the hippocampus ( Zhang *et al.*, 2012).

### Nociceptive behaviors are not impaired in *Entpd3 ^-/-^* mice

**Figure 11.**
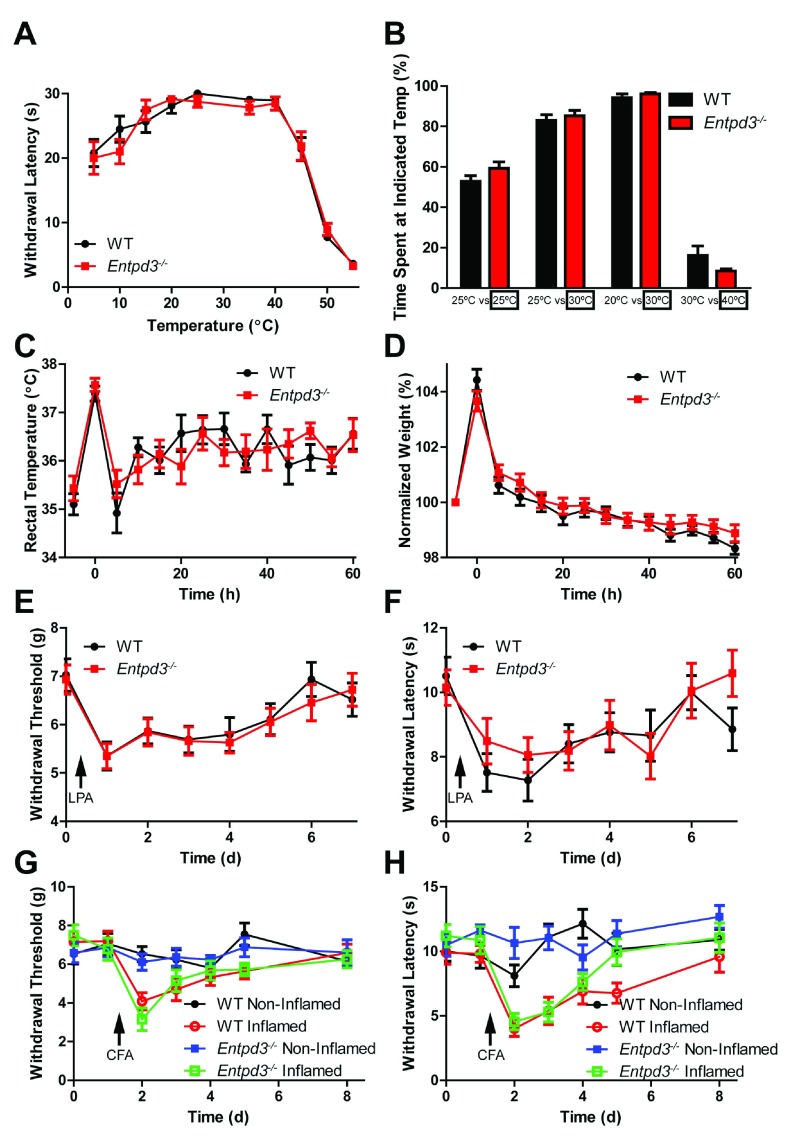
*Entpd3 ^-/-^* mice show no nociceptive or thermoregulatory behavioral deficits. ( **A**) Sensitivity to temperatures ranging from noxious cold to noxious hot was measured using the hindpaw withdrawal assay. Cutoff time was 30 s. ( **B**) Two-choice temperature discrimination assay. Temperatures were maintained at 25°C/25°C, 25°C/30°C, 20°C/30°C, or 30°C/40°C, and time on each side was measured for 10 minute. ( **C** and **D**) The water repulsion assay examined rectal body temperature and body weight before and after immersion into a 37°C water bath for 2 min. ( **E**– **H**) Mechanical allodynia and thermal hyperalgesia were measured in the LPA model of neuropathic pain ( **E** and **F**) and in the CFA model of inflammatory pain ( **G** and **H**). n=10 per group. t-tests were used to compare responses between genotypes at each time point, no significant differences detected. All values are represented as means ± SEM.

Given the high expression of ENTPD3 in nociceptive neurons, we examined whether loss of ENTPD3 affected nociceptive-related behaviors by testing heat, cold, mechanical, and itch sensation ( Table 3). In tests of heat sensitivity, there was no difference between WT and *Entpd3 ^-/-^* mice in the tail immersion assay (46.5°C or 49°C; Table 3). Similarly, there was no difference in withdrawal latency in the hot plate test ( Table 3). There was also no difference in responses between WT and *Entpd3* ^-/-^ mice in any of the cold assays (acetone evaporative cooling, cold tail immersion at -10°C, or cold plantar; Table 3). To further validate our thermal data, we used a hindpaw withdrawal assay ( Gentry *et al.*, 2010) that measures sensitivity to temperatures ranging from noxious cold to noxious hot ( Figure 11A). No difference was found between WT and *Entpd3 ^-/-^* mice at any temperature. We also examined responses to mechanical stimuli and observed no difference between WT and *Entpd3 ^-/-^* mice in noxious mechanical (tail clip) and innocuous mechanical (cotton swab) assays ( Table 3).

**Table 3.** Quantification of noxious heat-related, itch, and cold behavior assays.

Behavior	Response	P Value
***HEAT***		
**Tail Immersion (46.5°C)**	Latency to Flick (s)	
Saline	28.0 ± 1.8	0.18
*Entpd3 ^-/-^*	30.6 ± 2.1	
**Tail Immersion (49°C)**	Latency to Flick (s)	
Saline	8.6 ± 0.7	0.47
*Entpd3 ^-/-^*	8.7 ± 1.0	
**Hot Plate (52°C)**	Withdrawal Latency (s)	
Saline	30.2 ± 2.6	0.19
*Entpd3 ^-/-^*	26.3 ± 3.5	
***ITCH***		
**Histamine**	Scratching Bouts	
Saline	107.3 ± 14.2	0.18
*Entpd3 ^-/-^*	129.2 ± 18.5	
**Chloroquine**	Scratching Bouts	
Saline	260.2 ± 52.5	0.48
*Entpd3 ^-/-^*	263.0 ± 38.3	
**β-Alanine**	Scratching Bouts	
Saline	33.3 ± 5.6	0.04
*Entpd3 ^-/-^*	21.9 ± 2.5*	
***COLD***		
**Acetone**	Time Spent Licking (s)	
Saline	2.6 ± 0.7	0.19
*Entpd3 ^-/-^*	1.9 ± 0.4	
**Tail Immersion (-10°C)**	Latency to Flick (s)	
Saline	49.2 ± 3.2	0.24
*Entpd3 ^-/-^*	52.2 ± 2.7	
**Cold Plantar**	Withdrawal Latency (s)	
Saline	11.7 ± 0.5	0.39
*Entpd3 ^-/-^*	11.5 ± 0.4	
***MECHANICAL***		
**Cotton Swab**	Withdrawal Frequency (%)	
Saline	47.0 ± 8.5	0.43
*Entpd3 ^-/-^*	49.0 ± 6.6	
**Tail Clip**	Latency to Bite Clip (s)	
Saline	8.2 ± 1.3	0.20
*Entpd3 ^-/-^*	6.9 ± 0.7	

n = 10 mice/group, *p < 0.05.

To determine if loss of ENTPD3 affected itch, we injected pruritogens (histamine, chloroquine, β-alanine) into the nape of the neck and quantified scratching responses. Histamine- and chloroquine-mediated itch were not altered in *Entpd3 ^-/-^* mice compared to WT mice, but there was a statistically significant reduction (a decrease of 34%) in β-alanine-mediated itch ( Table 3). β-alanine activates the Mas-related G-protein-coupled receptor D (MRGPRD) in nonpeptidergic nociceptive neurons ( Liu *et al.*, 2012; Rau *et al.*, 2009; Shinohara *et al.*, 2004). Therefore, it is possible that loss of ENTPD3 affects nonpeptidergic DRG neurons. When taken together, these data suggest that ENTPD3 does not play a widespread role in regulating sensitivity to noxious or innocuous somatosensory stimuli.

### Temperature discrimination and thermoregulation are not impaired in *Entpd3 ^-/-^* mice

We next tested WT and *Entpd3 ^-/-^* mice in a two-temperature discrimination assay. In this assay, the amount of time spent in chambers with equal or different floor temperatures is quantified. Four temperature pairs were evaluated (25°C versus 25°C, 25°C versus 30°C, 20°C versus 30°C, and 30°C versus 40°C). There were no significant differences between WT and *Entpd3 ^-/-^* mice at any of the tested temperature pairs ( Figure 11B). These data indicate that temperature discrimination is not impaired in *Entpd3 ^-/-^* mice.

We next examined the extent to which *Entpd3 ^-/-^* mice regulate body temperature in the water repulsion assay. Mice were placed in a 37°C water bath for 2 minutes and their core body (rectal) temperatures and body weights were measured every 5 minutes for 60 minutes after removal from the water bath ( Figure 11C,D). Following removal from the water bath, WT and *Entpd3 ^-/-^* mice showed no differences in the initial body temperature increase or in the subsequent rate to recover their body temperature following hypothermia ( Figure 11C). These data demonstrate that *Entpd3 ^-/-^* mice have no deficits in body temperature regulation due to evaporative cooling.

The water repulsion assay also tests fur barrier function ( Westerberg *et al.*, 2004). Once the mouse is removed from the water bath, the initial increase in body weight is indicative of the amount of water absorbed by the fur. We found no significant difference between WT and *Entpd3 ^-/-^* mice in this assay ( Figure 11D), including in the rate at which water is removed/evaporates from the mice.

### Hyperalgesia and allodynia in *Entpd3 ^-/-^* mice are not impaired in models of chronic pain

Lastly, we sought to determine if deletion of ENTPD3 affected the magnitude of allodynia and hyperalgesia in models of inflammatory pain and neuropathic pain. Lysophosphatidic acid (LPA) is a pronociceptive ligand that sensitizes nociceptors and produces a chemically-induced form of neuropathic pain when injected intrathecally (i.t.) ( Inoue *et al.*, 2004). Administration of CFA into the hindpaw causes thermal hyperalgesia and mechanical allodynia and serves as a model of inflammatory pain. We monitored thermal and mechanical sensitivity before and after administration of either LPA (i.t.) or CFA (into hindpaw) and observed no differences between WT and *Entpd3 ^-/-^* mice in either chronic pain model ( Figure 11E,F).

## Conclusions

We generated a mouse that globally lacks ENTPD3 to evaluate the extent to which ENTPD3 was necessary for normal extracellular nucleotide hydrolysis in primary somatosensory neurons and dorsal spinal cord. Despite being expressed at high levels in many nociceptive and non-nociceptive somatosensory neurons, deletion of ENTPD3 did not affect extracellular nucleotide hydrolysis. Further, there were no changes in nociceptive behaviors in *Entpd3 ^-/-^* mice, though we did observe a small reduction in β-alanine-mediated itch response in knockout animals. These findings suggest that other enzymes are present that dephosphorylate extracellular nucleoside di- and triphosphates in primary somatosensory neurons. Our use of inhibitors ruled out the possibility that some ENTPDs, alkaline phosphatases and Na/K-ATPase compensated for the loss of ENTPD3. However, we cannot exclude the possibility that additional known or unknown enzymes with ectonucleotidase activity might be upregulated in *Entpd3 ^-/-^* mice and compensate for the loss of ENTPD3. Determining which enzymes act redundantly with ENTPD3 will require use of additional inhibitors and additional ectonucleotidase knockout lines. While ENTPD3 may function redundantly with other ectonucleotidases in these neurons, our *Entpd3* knockout line could prove useful in determining the physiological role of ENTPD3 in other organ systems where this ectonucleotidase is expressed, including in neurons that control wakefulness and feeding behavior ( Appelbaum *et al.*, 2007; Belcher *et al.*, 2006; Kiss *et al.*, 2009), in the cochlea ( Vlajkovic *et al.*, 2006), in cells that regulate insulin secretion ( Lavoie *et al.*, 2010; Syed *et al.*, 2013), and in the gastrointestinal system ( Lavoie *et al.*, 2011).

## Data availability

*F1000Research*: Dataset 1. Influence of ENTPD3 deletion on nucleotide hydrolysis in mouse primary somatosensory neurons and spinal cord: data, http://dx.doi.org/10.5256/f1000research.4563.d31211 ( McCoy *et al.*, 2014).
