# Supplementary material for: Deletion of ENTPD3 does not impair nucleotide hydrolysis in primary somatosensory neurons or spinal cord
Source: F1000Res. 2014 Sep 19;3:163. Originally published 2014 Jul 21. [Version 2] doi: 10.12688/f1000research.4563.2 (PMC4329602; doi:10.12688/f1000research.4563.2)
Supplement: Influence of ENTPD3 deletion on nucleotide hydrolysis in mouse primary somatosensory neurons and spinal cord: data — Please see the ‘Legends’ file in the zip file for descriptions of each data set. [file f1000research-3-5673-s0000.tgz › 87f052ab-5eac-4fb2-8244-f9c50a279e2b_ikmzx.zylkaf1000.zip!/Legends for F1000 data files (Bonnie and Eric and Martin).docx]

**Counts of ENTPD3+NF200 in WT DRGs (B12p108)**: This data represents the degree to which ENTPD3 and Neurofilament-200 (NF200) colocalize in the dorsal root ganglia (DRGs) of 3 wild-type mice. Data analysis was performed in Excel.

**Counts of ENTPD3+PAP, ENTPD3+CGRP in WT DRGs (B11p19):** This data represents the degree to which ENTPD3 and Prostatic acid phosphatase (PAP) and, separately, ENTPD3 and Calcitonin gene-related peptide (CGRP) colocalize in the dorsal root ganglia (DRGs) of 3 wild-type mice. Data analysis was performed in Excel.

**Counts of NeuN+CGRP, NeuN+IB4 in WT, ENTPD3KO DRGs (B11p12)**: This data represents expression of Calcitonin gene-related peptide (CGRP) and, separately, the binding of Isolectin B4 (IB4) in dorsal root ganglia (DRGs) of 3 wild-type and 3 *Entpd3^-/-^* mice. Expression is reported with respect to the neuronal marker NeuN. Data analysis was performed in Excel.

**Counts of NeuN+ENTPD3 in WT DRGs (B12p108):** This data represents the degree to which ENTPD3 is expressed in dorsal root ganglia (DRGs) of 3 wild-type mice. Expression is reported with respect to the neuronal marker NeuN. Data analysis was performed in Excel.

**Counts of NeuN+NF200 in WT, ENTPD3KO DRGs (B12p108):** This data represents expression of Neurofilament-200 (NF200) in dorsal root ganglia (DRGs) of 3 wild-type and 3 *Entpd3^-/-^* mice. Expression is reported with respect to the neuronal marker NeuN. Data analysis was performed in Excel.

**Counts of NeuN+NT5E in WT, ENTPD3KO DRGs (B11p16):** This data represents expression of 5’-Ectonucleotidase (NT5E) in dorsal root ganglia (DRGs) of 3 wild-type and 3 *Entpd3^-/-^* mice. Expression is reported with respect to the neuronal marker NeuN. Data analysis was performed in Excel.

**Counts of NeuN+PAP in WT, ENTPD3KO DRGs (B11p25):** This data represents expression of Prostatic acid phosphatase (PAP) in dorsal root ganglia (DRGs) of 3 wild-type and 3 *Entpd3^-/-^* mice. Expression is reported with respect to the neuronal marker NeuN. Data analysis was performed in Excel.

**25v25 Temperature Preference Assay Behavior:** The data are displayed as the time each mouse spent at 25°C side A which was separated into minutes and seconds. The total time, in seconds, is added up and divided by the total time (600 min). The percent time is calculated for each side and the mean for each side is determined. Data analysis was performed in EXCEL and graphs were generated in GraphPad Prism.

**20v30 Temperature Preference Assay Behavior:** The data are displayed as the time each mouse spent at 30°C which was separated into minutes and seconds. The total time, in seconds, is added up and divided by the total time (600 min). The percent time is calculated for each side and the mean for each side is determined. Data analysis was performed in EXCEL and graphs were generated in GraphPad Prism.

**25v30 Temperature Preference Assay Behavior:** The data are displayed as the time each mouse spent at 30°C which was separated into minutes and seconds. The total time, in seconds, is added up and divided by the total time (600 min). The percent time is calculated for each side and the mean for each side is determined. Data analysis was performed in EXCEL and graphs were generated in GraphPad Prism.

**30v40 Temperature Preference Assay Behavior:** The data are displayed as the time each mouse spent at 30°C which was separated into minutes and seconds. The total time, in seconds, is added up and divided by the total time (600 min). The percent time is calculated for each side and the mean for each side is determined. Data analysis was performed in EXCEL and graphs were generated in GraphPad Prism.

**Acetone Behavior:** The data are displayed as the time each mouse spent licking its hindpaw following application of acetone for 1 min. Data analysis was performed in EXCEL and graphs were generated in GraphPad Prism. Mean time was calculated.

**Beta Alanine Behavior:** The data are displayed as the bouts of scratches for each mouse. Data is separated for each 5-min interval with the mean for each 5-min interval calculated and the total for each mouse calculated. Data analysis was performed in EXCEL and graphs were generated in GraphPad Prism.

**Chloroquine Behavior:** The data are displayed as the bouts of scratches for each mouse. Data is separated for each 5-min interval with the mean for each 5-min interval calculated and the total for each mouse calculated. Data analysis was performed in EXCEL and graphs were generated in GraphPad Prism.

**Cold Plantar Behavior:** The data are displayed as the amount of time it took for each mouse to withdraw its paw following application of a cold stimulus. Right and left paws were measured and averaged. Mean time was calculated. Data analysis was performed in EXCEL and graphs were generated in GraphPad Prism.

**Cotton Swab Behavior:** The data are shown as the number of flinches per total times the cotton swab was applied. This was then calculated as a percentage and then percentages were averaged for both paws and mean frequency was calculated. Data analysis was performed in EXCEL and graphs were generated in GraphPad Prism.

**Hargreaves CFA Behavior:** The data are displayed as the amount of time it took for each mouse to withdraw its paw following application of a radiant heat stimulus. Right and left paws were measured. Complete Freunds Adjuvant (CFA) was administered on day 1 after measurements were made. Mean time was calculated. Data analysis was performed in EXCEL and graphs were generated in GraphPad Prism. BL=baseline.

**Hargreaves LPA Behavior:** The data are displayed as the amount of time it took for each mouse to withdraw its paw following application of a radiant heat stimulus. Right and left paws were measured and combined. Lysophosphatidic acid (LPA) was administered on BL after measurements were made. Mean time was calculated. Data analysis was performed in EXCEL and graphs were generated in GraphPad Prism. BL=baseline.

**Hindpaw Withdrawal Assay Behavior:** The data are displayed as the amount of time it took for each mouse to withdraw its paw after it was placed onto a metal plate set at different temperatures. The mean was calculated for each temperature tested. Data analysis was performed in EXCEL and graphs were generated in GraphPad Prism.

**Histamine Behavior:** The data are displayed as the bouts of scratches for each mouse. Data is separated for each 5-min interval with the mean for each 5-min interval calculated and the total for each mouse calculated. Data analysis was performed in EXCEL and graphs were generated in GraphPad Prism.

**Hot Plate Behavior:** The data are displayed as the amount of time it took for each mouse to lift or flinch its hindpaw. Mean was calculated for each genotype. Data analysis was performed in EXCEL and graphs were generated in GraphPad Prism.

**Tail Clip Behavior:** The data are displayed as the amount of time it took for each mouse to bite the clip. Mean was calculated for each genotype. Data analysis was performed in EXCEL and graphs were generated in GraphPad Prism.

**Tail Immersion Behavior:** The data are displayed as the latency for the mice to flick their tail out of the water. Mean was calculated for each genotype and temperature. Data analysis was performed in EXCEL and graphs were generated in GraphPad Prism.

**Von Frey CFA Behavior:** The data are displayed as the amount of force it took for each mouse to withdraw its paw. Right and left paws were measured. Complete Freunds Adjuvant (CFA) was administered on day 1 after measurements were made. Mean time was calculated. Data analysis was performed in EXCEL and graphs were generated in GraphPad Prism. BL=baseline.

**Von Frey LPA Behavior:** The data are displayed as the amount of force it took for each mouse to withdraw its paw. Right and left paws were measured and combined. Lysophosphatidic acid (LPA) was administered on BL after measurements were made. Mean time was calculated. Data analysis was performed in EXCEL and graphs were generated in GraphPad Prism. BL=baseline.

**Water Repulsion Behavior Temperature:** The data are displayed as the rectal temperature recorded every 5 min for 60 min. Means were calculated at each time point. Data analysis was performed in EXCEL and graphs were generated in GraphPad Prism.

**Water Repulsion Behavior Weight:** The data are displayed as the body weight recorded every 5 min for 60 min. Means were calculated at each time point. Data were normalized to account for differences in initial body weight. Data analysis was performed in EXCEL and graphs were generated in GraphPad Prism.

# Fast-scan cyclic voltammetry:

# Description of analysis

The FSCV data were analyzed using the analysis portion of the freely available software HDCV (Version 4), which is detailed in Bucher et al.

The software is available for download from: http://www.chem.unc.edu/facilities/index.html?display=electronics&content=software

Default parameters were used for row and column averaging. No filters were used.

# Description of files

**Figure 9a WT** and **Figure 9a Entpd3 dKO (.tsv/without extension):** Numerical data corresponding to the Wild Type and *Entpd3^-/-^* color plots in figure 9a. Values are currents in nA.

.tsv are tab-separated text files. Files Without extension are suitable for opening in HDCV.

**Figure 9b.tsv**: Numerical data for figure 9b

**Figure 9c.tsv**: Numerical data for figure 9c. Note: the data extend in the file encompass a greater time range both before and after the ejection. The time range for the figure was chosen to show the relevant time portion. The conversion from current to concentration is performed as described in the text.

**Paired TTest values.tsv:** Average peak currents from the FSCV data that were used to perform the t-test. Rows represent the pairs.

**Sample dKO/WTnTrialxx.tsv/ Sample dKO/WTnTrialxx:** Raw FSCV data for the n^th^ *Entpd3^-/-^* or WT sample, ejection number xx. The .tsv files are tab-separatedASCII. The files without extension are suitable for opening directly in HDCV.

**Reference**

Bucher ES, Brooks K, Verber MD, Keithley RB, Owesson-White C, Carroll S, Takmakov P, McKinney CJ, Wightman RM. “Flexible software platform for fast-scan cyclic voltammetry data acquisition and analysis.” *Anal Chem*. 2013 Nov 5;85(21). 10344-53.
