# Supplementary material for: Deletion of ENTPD3 does not impair nucleotide hydrolysis in primary somatosensory neurons or spinal cord
Source: F1000Res. 2014 Sep 19;3:163. Originally published 2014 Jul 21. [Version 2] doi: 10.12688/f1000research.4563.2 (PMC4329602; doi:10.12688/f1000research.4563.2)
Supplement: Peer review history

# Peer review: Referee response for version 2

**DOI:** 10.5256/f1000research.5673.r6192

**Published:** 2014-11-06

**Authors:** Derek Molliver

**Affiliations:** 1 Department of Biomedical Sciences, University of New England, Portland, ME, USA

The authors have effectively addressed several reviewer concerns in the revised manuscript.

I have read this submission. I believe that I have an appropriate level of expertise to confirm that it is of an acceptable scientific standard.

# Peer review: Referee response for version 2

**DOI:** 10.5256/f1000research.5673.r6191

**Published:** 2014-10-01

**Authors:** Terry Kirley

**Affiliations:** 1 Department of Pharmacology and Cell Biophysics, University of Cincinnati, Cincinnati, OH, USA

The authors have adequately addressed my comments and concerns.

I have read this submission. I believe that I have an appropriate level of expertise to confirm that it is of an acceptable scientific standard.

# Peer review: Referee response for version 1

**DOI:** 10.5256/f1000research.4882.r5940

**Published:** 2014-08-28

**Authors:** Derek Molliver

**Affiliations:** 1 Department of Biomedical Sciences, University of New England, Portland, ME, USA

In this paper, McCoy *et al.* present novel results regarding the contribution of ENTPD3 to nucleotide hydrolysis in primary sensory neurons and the spinal cord. Immunohistochemical analysis of ENTPD3 distribution using a commercial antibody was highly similar to a previous report (full disclosure, from this reviewer’s laboratory) cited in the paper; selectivity of the antibody was effectively confirmed using tissue from knockout mice generated by the authors. The study is well-executed and the figures are outstanding.

The key findings were generated through functional analysis of the knockout mice, including nucleotidase histochemistry, extensive behavioral analysis of nociceptive and non-nociceptive thresholds in naïve and inflamed mice, and fast-scan cyclic voltammetry (FSCV), an innovative method for measuring adenosine levels in situ in spinal cord slices. These studies found that ENTPD3 was dispensible for nucleotide hydrolysis, and all measured behavioral variables were unaltered in knockout mice, with the exception of a reduction in b-alanine-mediated itch behavior. The results suggest the possibility that additional ectonucleotidase(s) are co-expressed with ENTPD3 that are sufficient for normal nucleotide triphosphate/diphosphate hydrolysis, similar to the situation described by these authors for the ectonucleotidases that generate adenosine from AMP. The histological results support the conclusion that ENTPD3 is in position to impact both noxious and non-noxious somosensory transduction and transmission, but underscore that the regulation of somatosensory purinergic signaling is complex and likely to be regulated by multiple enzymes acting in tandem. This is a significant finding for the pain field, because ENTPD3 is the only ectonucleotidase identified in primary sensory neurons that regulates the availability of extracellular ATP and UTP, which have been extensively implicated in nociceptive signaling as agonists for P2X and P2Y receptors.

A key point not fully addressed here is whether knockout of ENTPD3 results in upregulation of other NTPDases in DRG neurons and/or dorsal horn, which could provide an explanation for the mild knockout phenotype. In particular, analysis of neuronal mRNA/ protein levels and distribution for ENTPD1, 2 and 8 in ENTPD3 knockout mice would increase the impact of the findings reported here. An intriguing possibility is that the alternate enzymes responsible are not members of the ENTPD family. The authors do demonstrate that the ENTPD1 inhibitor ARL67156 did not alter the distribution of enzyme histochemical staining in knockout tissue compared to WT, but  did eliminate microglial labeling in both genotypes.

One question that the authors might want to address in the discussion is how to evaluate whether FSCV is capable of resolving neuronal ENTPD3 activity in the dorsal horn when the neurons are surrounded by microglia expressing ENTPD1 (the active site of these enzymes is extracellular). As the authors suggest, further analysis in mice with multiple ENTPD gene deletions may be informative. However, the substantial behavioral evidence indicates that loss of ENTPD3 is not critical for normal sensory processing.

I have read this submission. I believe that I have an appropriate level of expertise to confirm that it is of an acceptable scientific standard.

# Response

**Published:** 2014-09-11

**Authors:** Mark Zylka

**Affiliations:** The University of North Carolina at Chapel Hill, USA

We thank Dr. Molliver for his comments, particularly given his expertise in studying ENTPD3 in sensory neurons.  As Dr. Molliver notes, we attempted to address this issue of compensation using a number of inhibitors in combination with Entpd3-/- tissues.

Drs. Kirley and Molliver raised the question of whether other ectonucleotidases might be upregulated and compensate for the loss of ENTPD3.  We understand the rationale and interest behind this comment.  At this time, we feel such experiments are beyond the scope and focus of our initial study for the following reasons:

1. If we were to find that other ecto-enzymes were upregulated, this information alone would not prove that any upregulated enzymes compensate or act redundantly in the absence of Entpd3.
2. There are numerous ectonucleotidases (NTPDases, NPPs, alkaline phosphatases, acid phosphatases, Na/K-ATPases and likely additional enzymes with ectonucleotidase activity) that could be altered upon Entpd3 deletion.  We thus may miss the most relevant enzyme (or enzymes) if we focus on a candidate list.  Indeed, our attempt to focus on several candidates, via our use of inhibitors, was unsuccessful, suggesting this is a complex issue with multiple enzymes involved.   A genome-wide expression analysis would be more comprehensive but would not provide insights as to which upregulated genes are biologically relevant—knowing what genes change will not allow us to prove they compensate for the loss of Entpd3.
3. Our study was not focused on examining potential compensatory mechanisms in the Enptd3-/- mice.  Such a study will require substantial effort to do properly (i.e. we would first need to identify all the enzymes that are upregulated and then demonstrate, in a systematic manner, that each one does or does not act redundantly using inhibitors and/or double/triple knockout mice).  Such an endeavor would require considerable effort. 

Thus, in lieu of examining a candidate list of ectonucleotidases, we felt a compromise would be to address this comment as follows (by revising the Conclusions section):

*“Our use of inhibitors ruled out the possibility that some ENTPDs, alkaline phosphatases and Na/K-ATPase compensated for the loss of ENTPD3.  However, we cannot exclude the possibility that additional known or unknown enzymes with ectonucleotidase activity might be upregulated in Entpd3-/- mice and compensate for the loss of ENTPD3.  Determining which enzymes act redundantly with ENTPD3 will require use of additional inhibitors and additional ectonucleotidase knockout lines.”*

  

**Dr Molliver: **"One question that the authors might want to address in the discussion is how to evaluate whether FSCV is capable of resolving neuronal ENTPD3 activity in the dorsal horn when the neurons are surrounded by microglia expressing ENTPD1 (the active site of these enzymes is extracellular). As the authors suggest, further analysis in mice with multiple ENTPD gene deletions may be informative. However, the substantial behavioral evidence indicates that loss of ENTPD3 is not critical for normal sensory processing."

To address this comment, we added the following sentence to the Results & Discussion section:

*“Note that FSCV cannot resolve neuronal ENTPD3 activity in the dorsal horn from spinal microglial ENTPD1 activity, so the adenosine detected by FSCV after applying ADP could originate from microglial ENTPD1 or other ectonucleotidases in the tissue.  For example, this adenosine could originate from PAP and/or TNAP, as these enzymes are located in the same region and can also hydrolyze ADP to adenosine (Figure 1).”*

# Peer review: Referee response for version 1

**DOI:** 10.5256/f1000research.4882.r5504

**Published:** 2014-08-01

**Authors:** Terry Kirley

**Affiliations:** 1 Department of Pharmacology and Cell Biophysics, University of Cincinnati, Cincinnati, OH, USA

The paper by McCoy *at al.* entitled “ *Deletion of ENTPD3 does not impair nucleotide hydrolysis in primary somatosensory neurons or spinal cord*” is a detailed and technically sound study. This paper focuses on possible roles for ENTPD3/NTPDase3 in pain perception and neural transmission in the dorsal root ganglia (DRG) and spinal cord. In addition, other proteins of interest that are related and known to be involved in the DRG, including prosthetic acid phosphatase (PAP), 5’-nucleotidase (NT5E) and tissue nonspecific acid phosphatase (TNAP) were examined. Many behavioral and pain assays were performed. These include tail immersion assays, a hot plate test, tail clip assay, cotton swab assay, acetone test, cold plantar assay, and 2-temperature discrimination assays. In addition, several assays for itch were performed, including histamine, chloroquine, and beta-alanine induced itch. Also, several other behavioral assays, including the water repulsion assay, the Complete Freund’s Adjuvant (CFA) inflammation pain assay and the LysoPhosphatidic Acid (LPA) neuropathic pain assays were performed.

In general, the work is well done and detailed, and includes the appropriate controls. However, the main problem with this work is that there is no indication of the physiological function of NTPDase3/ENTPD3 revealed by any of the experimental results. Of course, negative data is sometimes important, and this is so in this case. However, what is really missing from this work is analysis for other nucleotidases that are likely to compensate for the loss of NTPDase3 in these mice. These include the nucleotide pyrophosphatase/phosphodiesterase enzymes (NPPs), and more importantly, the other members of the cell-surface NTPDase class of nucleotidases, especially NTPDase1/ENTPD1, and NTPDase2/ENTPD2.  Since the authors claim in their abstract that “ *there could be multiple ectonucleotidases that act redundantly to hydrolyze nucleotides in these regions of the nervous system*” (which seems logical and likely), it is somewhat curious that no analyses for these other nucleotidases were performed. If such experiments were done, and if up-regulation of one or more of these enzymes was observed, this study would be more interesting, and the paper more important, since this might suggest putative physiological role(s) for NTPDase3/ENTPD1.

There are a couple of interesting and possibly problematic experimental details reported in the paper. First, why was 20 mM magnesium chloride used in the enzyme histochemistry experiments? This seems to be an unreasonably high, non-physiologic, concentration of magnesium. In addition, many of these nucleotidases, including NTPDase3 and other NTPDases, are, in fact, more active using calcium as a divalent cation as opposed to magnesium. So the choice of 20 mM magnesium chloride seems odd. Also, one change in these knockout mice that is noted in terms of possible effects on nucleotide hydrolysis is the decrease in 5’-nucleotidase protein seen in the DRG neurons of the knockout mouse, which is reported in Table 2. However, as reported in Figure 7, there is no apparent decrease in hydrolysis of AMP in the same dorsal root ganglia, which is apparently not consistent with Table 2, although other enzymes could come into play (but don’t seem to change in the KO). However, enzyme histochemistry is difficult to accurately quantitate and is usually regarded as a semi-quantitative technique. Thus, a relatively small change in hydrolysis rates may not be evident from enzyme histochemical data. This is a potential problem with Figure 7, and begs the question as to why tissue homogenates were not evaluated by solution-based, quantitative nucleotidase enzyme assays. The same limitations are applicable to the data reported in Figure 8 on spinal cord sections from wild type and knockout mice. As reported in Table 3, the authors did find a significant difference in itch response to beta-alanine in the KO mice. However, again, the other itch data, and the rest of the data in Table 3, show no difference between wt and KO mice for responses to itch, heat, or cold behavioral stimuli.

In their conclusion section, the authors do mention other roles that have been suggested for NTPDase3/ENTPD3, including possible roles in the hypothalamus for controlling wakefulness and feeding behavior, for hearing in the cochlea, in the beta cells of the pancreas for regulation of ATP-controlled insulin secretion, and in the G.I. tract. It would be interesting to report any experiments designed to monitor for changes in any of these putative physiological functions of NTPDase3. These could include measurements designed to detect abnormal sleep times or cycles, abnormal eating habits, and abnormal plasma glucose and insulin levels and/or abnormal responses to glucose tolerance tests.

In conclusion, this study is well done and thorough with respect to the attributes that were evaluated in the DRG and spinal cord. Unfortunately, the results do not suggest likely physiological function(s) for NTPDase3/ENTPD3. Also, there is no data reported for other related cell-surface nucleotidases, such as NTPDases 1 and 2, which may be up-regulated in a compensatory response to the knockout of NTPDase3. In addition, there is no mention of experiments designed to address other putative physiological functions of NTPDase3, which are not related to the DRG or spinal cord. Hopefully, these points will be addressed in future work on these knockout animals.

I have read this submission. I believe that I have an appropriate level of expertise to confirm that it is of an acceptable scientific standard.

# Response

**Published:** 2014-09-11

**Authors:** Mark Zylka

**Affiliations:** The University of North Carolina at Chapel Hill, USA

We thank Dr. Kirley for his comments and for appreciating that our study, which is focused on Entpd3 in sensory neurons and spinal cord, largely reports negative findings.  This marks the first reported Entpd3 knockout mouse.  A priori, it was unknown if this ectonucleotidase would affect nucleotide hydrolysis in sensory neurons or spinal cord independent of the other ectonucleotidases that are present in these tissues. 

Since deletion of Entpd3 had no effect on nucleotide hydrolysis, we performed additional analyses to determine if other ectonucleotidases were involved.  This includes assessing whether alkaline phosphatases act redundantly with Entpd3 (using the inhibitor levamisole), assessing whether other Entpds act redundantly with Entpd3 (using the nonselective Entpd inhibitor ARL67156), and assessing whether Na/K-ATPases act redundantly with Entpd3 (using ouabain to inhibit these ATPases).  The outcomes of these experiments are described in the results section.

Drs. Kirley and Molliver raised the question of whether other ectonucleotidases might be upregulated and compensate for the loss of ENTPD3.  We understand the rationale and interest behind this comment.  At this time, we feel such experiments are beyond the scope and focus of our initial study for the following reasons:

1. If we were to find that other ecto-enzymes were upregulated, this information alone would not prove that any upregulated enzymes compensate or act redundantly in the absence of Entpd3.
2. There are numerous ectonucleotidases (NTPDases, NPPs, alkaline phosphatases, acid phosphatases, Na/K-ATPases and likely additional enzymes with ectonucleotidase activity) that could be altered upon Entpd3 deletion. We thus may miss the most relevant enzyme (or enzymes) if we focus on a candidate list.  Indeed, our attempt to focus on several candidates, via our use of inhibitors, was unsuccessful, suggesting this is a complex issue with multiple enzymes involved.   A genome-wide expression analysis would be more comprehensive but would not provide insights as to which upregulated genes are biologically relevant—knowing what genes change will not allow us to prove they compensate for the loss of Entpd3.
3. Our study was not focused on examining potential compensatory mechanisms in the Enptd3-/- mice.  Such a study will require substantial effort to do properly (i.e. we would first need to identify all the enzymes that are upregulated and then demonstrate, in a systematic manner, that each one does or does not act redundantly using inhibitors and/or double/triple knockout mice).  Such an endeavor would require considerable effort.  For example, it took us several years and multiple knockout lines to rigorously demonstrate that PAP, NT5E and TNAP act redundantly to generate adenosine from AMP.

 Thus, in lieu of examining a candidate list of ectonucleotidases, we felt a compromise would be to address this comment as follows (by revising the Conclusions section):

*“Our use of inhibitors ruled out the possibility that some ENTPDs, alkaline phosphatases and Na/K-ATPase compensated for the loss of ENTPD3.  However, we cannot exclude the possibility that additional known or unknown enzymes with ectonucleotidase activity might be upregulated in Entpd3-/- mice and compensate for the loss of ENTPD3.  Determining which enzymes act redundantly with ENTPD3 will require use of additional inhibitors and additional ectonucleotidase knockout lines.”*

**Dr Kirley:** "There are a couple of interesting and possibly problematic experimental details reported in the paper. First, why was 20 mM magnesium chloride used in the enzyme histochemistry experiments? This seems to be an unreasonably high, non-physiologic, concentration of magnesium. In addition, many of these nucleotidases, including NTPDase3 and other NTPDases, are, in fact, more active using calcium as a divalent cation as opposed to magnesium. So the choice of 20 mM magnesium chloride seems odd."

To address this comment, we performed new experiments.  We performed histochemistry experiments with 2 mM CaCl _2_, 20 mM CaCl _2_ and 20 mM MgCl _2_, in WT and Entpd3-/- mice.  These data are shown in new Figure 9.

We also updated the results to include this new information:

*“Many ectonucleotidases, including ENTPD3 (Lavoie et al reference: PMID: 15130768; currently ref #23 “Cloning and characterization of mouse nucleoside triphosphate diphosphohydrolase-3”), are slightly more active in biochemical assays with calcium as the divalent cation.  However, we detected no difference in UTP histochemical activity in spinal cord between WT and Entpd3-/- mice when 2 mM or 20 mM CaCl _2 _was substituted for 20 mM MgCl _2_ (Figure 9; with deletion of ENTPD3 confirmed in these sections using immunostaining, Figure 9H).  Thus Mg ^2+ ^and Ca ^2+^ appear to be interchangeable in this histochemical assay.”*

Also please note, we used 20 mM MgCl in our histochemical experiments because a previous study, which we based our histochemistry method on, found that ATP ectonucleotidase activity in skin Langerhans cells was divavelent cation dependent, with complete interchangability between Ca ^2+^ and Mg ^2+^, and with optimal staining at a 20 mM concentration of either divalent (see Chaker *et al.*, 1984).  20 mM MgCl _2_ or 20 mM CaCl _2_ thus appears to be optimal for ATP histochemical staining.  And in biochemical assays with ENTPDs, Mg ^2+^ and Ca ^2+^ were interchangeable when ATP and ADP were used as substrates ( Rucker *et al.*, 2008).

**Dr Kirley: **"Also, one change in these knockout mice that is noted in terms of possible effects on nucleotide hydrolysis is the decrease in 5’-nucleotidase protein seen in the DRG neurons of the knockout mouse, which is reported in Table 2. However, as reported in Figure 7, there is no apparent decrease in hydrolysis of AMP in the same dorsal root ganglia, which is apparently not consistent with Table 2, although other enzymes could come into play (but don’t seem to change in the KO). However, enzyme histochemistry is difficult to accurately quantitate and is usually regarded as a semi-quantitative technique. Thus, a relatively small change in hydrolysis rates may not be evident from enzyme histochemical data. This is a potential problem with Figure 7, and begs the question as to why tissue homogenates were not evaluated by solution-based, quantitative nucleotidase enzyme assays. The same limitations are applicable to the data reported in Figure 8 on spinal cord sections from wild type and knockout mice."

We agree that histochemical staining provides a semi-quantitative readout of enzyme activity.  This is why we turned to FSCV in spinal cord slices.  FSCV provides a quantitative electrochemical method for measuring hydrolysis of ADP to adenosine, in the precise anatomical region where Entpd3 is located.  Since we found no differences between WT and Entpd3-/- mice using this quantitative electrochemical technique, we feel these data are sufficient to show that loss of Entpd3 alone has no measurable effect on nucleotide hydrolysis. 

And, as can be seen from our micrographs, Entpd3 is restricted to the dorsal spinal cord while ATP and ADP histochemical activity is broadly distributed. Use of a solution-based assay would entail creating homogenates from spinal cord or DRG, thus disrupting the integrity of the tissue and introducing more ectonucleotidases into the assay (which would reduce signal-to-noise).

The small 4.5% reduction in NT5E in DRG was statistically significant, although it appears to have no effect on AMP hydrolysis, as assessed histochemically.  This likely reflects that AMP can be hydrolyzed by NT5E, PAP and TNAP, as we previously found.  Since this did not constitute a major finding, we did not focus or discuss this in the text. 

**Dr Kirley: "**As reported in Table 3, the authors did find a significant difference in itch response to beta-alanine in the KO mice. However, again, the other itch data, and the rest of the data in Table 3, show no difference between wt and KO mice for responses to itch, heat, or cold behavioral stimuli."

We felt it would be difficult to experimentally pursue the mechanistic basis for this itch phenotype because it was extremely small in magnitude.  Such small behavioral effects are not easy to pursue.  Moreover, it was the only sensory phenotype out of a large number of sensory functions we probed, suggesting it is a very mild sensory phenotype.

**Dr Kirley: "**In their conclusion section, the authors do mention other roles that have been suggested for NTPDase3/ENTPD3, including possible roles in the hypothalamus for controlling wakefulness and feeding behavior, for hearing in the cochlea, in the beta cells of the pancreas for regulation of ATP-controlled insulin secretion, and in the G.I. tract. It would be interesting to report any experiments designed to monitor for changes in any of these putative physiological functions of NTPDase3. These could include measurements designed to detect abnormal sleep times or cycles, abnormal eating habits, and abnormal plasma glucose and insulin levels and/or abnormal responses to glucose tolerance tests."

These are indeed interesting topics for future study.  However, we feel they are beyond scope of our present study, which is focused on examining the function of Entpd3 in primary somatosensory neurons and dorsal spinal cord.

**Dr Kirley: **"In conclusion, this study is well done and thorough with respect to the attributes that were evaluated in the DRG and spinal cord. Unfortunately, the results do not suggest likely physiological function(s) for NTPDase3/ENTPD3. Also, there is no data reported for other related cell-surface nucleotidases, such as NTPDases 1 and 2, which may be up-regulated in a compensatory response to the knockout of NTPDase3. In addition, there is no mention of experiments designed to address other putative physiological functions of NTPDase3, which are not related to the DRG or spinal cord. Hopefully, these points will be addressed in future work on these knockout animals."

We agree that future studies are warranted.  By reporting our findings with these first ever Entpd3 knockout mice, it will now be possible for us and others to study Entpd3 in other physiological contexts and to explore possible compensatory mechanisms.
